# Characterization of the complete chloroplast genome of *Malus spectabilis* ‘Guanghui’

**DOI:** 10.1080/23802359.2020.1780982

**Published:** 2020-06-24

**Authors:** Qiqing Jiao, Jihan Tao, Chuanzeng Wang, Yanlei Yin, Lijuan Feng

**Affiliations:** a Shandong Institute of Pomology, Taian, China; b Shandong Academy of Agricultural Sciences, Jinan, China

**Keywords:** *Malus spectabilis* ‘Guanghui’, lllumina sequencing, chloroplast genome, phylogenetic relationship

## Abstract

*Malus spectabilis* ‘Guanghui’ is an important ornamental plant, which complete chloroplast genome (Accession: MT501657) was sequenced, assembled and annotated. The genome size is 1601,230 bp and the overall GC content is 36.50%, with large single-copy (LSC, 89,310bp) regions, small single-copy (SSC, 19,196 bp) regions, and two inverted repeat regions (IRs, 23,632bp each). A total of 129 genes are successfully annotated, including 84 protein-coding genes, 37 tRNA genes, and 8 rRNA genes. The phylogenetic relationships showed that *Malus spectabilis* ‘Guanghui’ is closely related to the species of *Malus sieversii*.

*Malus spectabilis* ‘Guanghui’ is an ornamental plant in garden and courtyard, which belongs to the *Malus* of Rosaceae family. Its tree is big, erect and strong, beautiful plant type. The color of new leaves is purple-red. The flower is red or fuchsia in May, and the fruit is red from July to October (An et al. 2018). The complete chloroplast (cp) genome is beneficial to reveal the evolutionary relationship of valuable species (Cai et al. 2020). In this study, we established and characterized the complete chloroplast (cp) genome of *Malus spectabilis* ‘Guanghui’ and provide additional effective data for the genetics conservation and phylogenetic study of *Malus* genus in the future.

The fresh leaves of *M. spectabilis* ‘Guanghui’ (Voucher specimen Accession No. SDGHHT0026) was collected from the Taidong field of Shandong Institute of Pomology (36.21°N, 117.12°E), Shandong Province, China. Total genomic DNA was extracted using the DNeasy Plant Mini Kit (Qiagen, Venlo, Netherlands). cpDNA sequencing were performed with an Illumina Hiseq 2500 platform by Nanjing Genepioneer Biotechnologies. The fastp program was used to filter the raw paired-end reads of cpDNA (Chen et al. 2018). The GetOrganelle was used to perform *de novo* assembly (Jin et al. 2018). The cp genome was annotated using the program DOGMA v1.2 (Wyman et al. 2004). The OGDRAW was used to generate the circular genome map of the genome (Lohse et al. 2013). The complete cp genome of *M. spectabilis* ‘Guanghui’ was deposited in the GenBank (Accession: MT501657).

The complete cp genome of *M. spectabilis* ‘Guanghui’ was 160,230 bp in length with overall GC content of 36.50%, exhibiting a typical four-conjoined structure. The cp genome is made up of large single-copy region (LSC) with 88,310 bp, small single-copy region (SSC) with 19,196 bp, and two inverted repeat regions (IRs) with 23,632 bp. The whole complete cp genome encoded 129 unique genes, which contained 84 protein-coding genes, 37 transfer RNA (tRNA) genes, and 8 ribosomal RNA (rRNA) genes. The tRNA genes are distributed throughout the whole genome with 22 in the LSC, 1 in the SSC, and 14 in the IR regions, while rRNAs are only situated in the IR regions. There were 18 duplicated genes in IRs, including 7 protein-coding genes, 7 tRNA genes, and 4 rRNA genes. Among the protein-coding genes, 3 genes (clpP, rps12, ycf3) contained two introns, and 9 genes (atpF, ndhA, ndhB, petB, petD, rp116, rpl2, rpoC1, rps16) possessed a single intron, and 6 tRNA genes (trnA-UGC, trnG-UCC, trnI-GAU, trnK-UUU, trnL-UAA, trnV-UAC) also had one intron.

To ascertain the phylogenetic position of *M. spectabilis* ‘Guanghui’, 18 complete cp genomes within Rosaceae family were selected. *Juglans regia* (GenBank Accession No. MF167463.1) and *Punica granatum* (GenBank Accession No. KY635883.1) were as outgroup. The maximum likelihood (ML) phylogenetic tree was constructed by the IQ-TREE with the best-fit model identified using ModelFinder (Kalyaanamoorthy et al. 2017). The result showed that *M. spectabilis* ‘Guanghui’ is closely related to the species of *M. sieversii* (Figure.1). It will be valuable for the genetic study on the *Malus* genus in Rosaceae.

**Figure 1.**
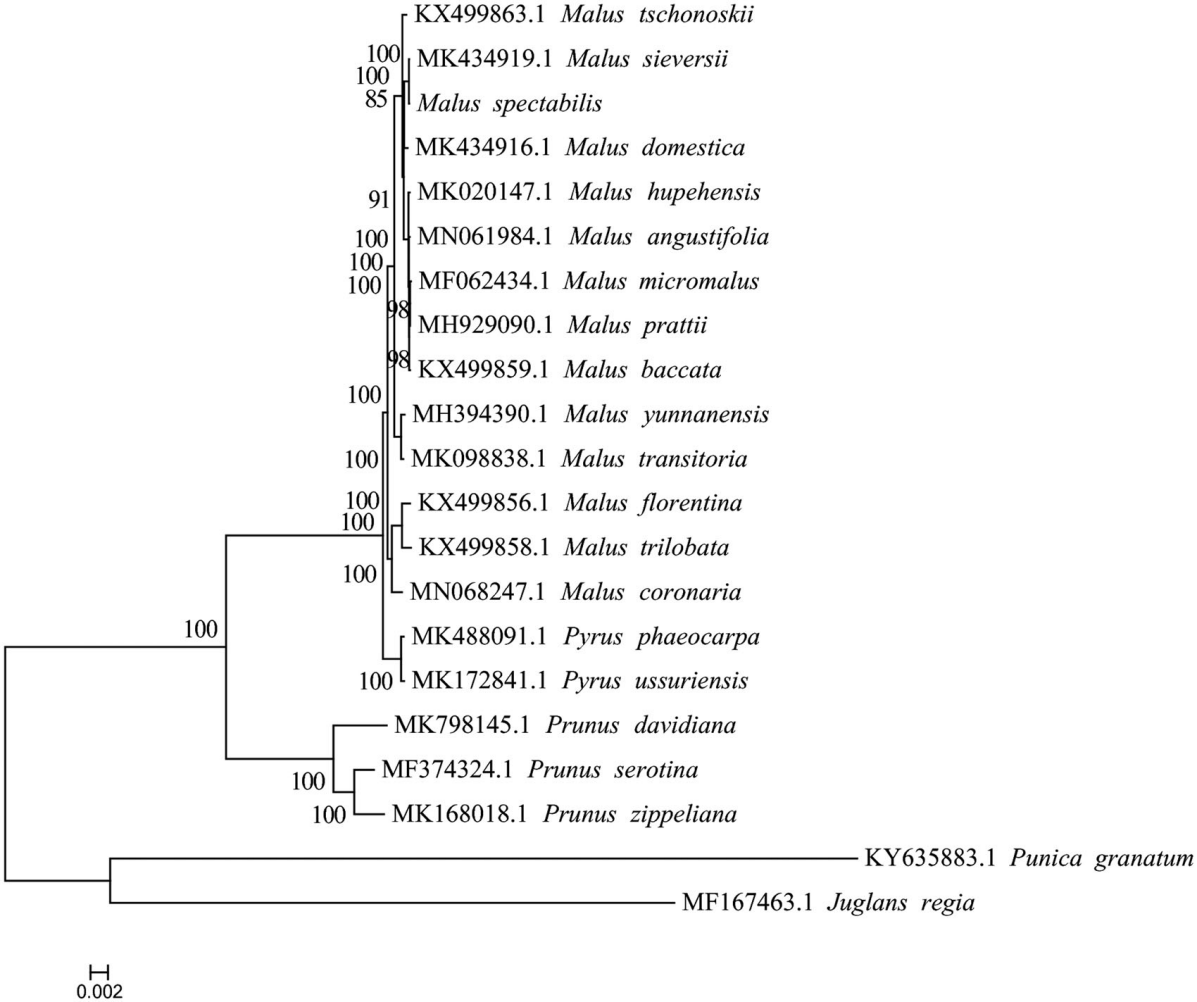
The phylogenetic tree of *M. spectabilis* ‘Guanghui’ and its related relatives based on complete chloroplast genome sequences. The number on each node indicates bootstrap support value.

## Data Availability

The data that support the findings of this study are openly available in the NCBI at https://www.ncbi.nlm.nih.gov/, reference number: MT501657.
